# Tumor-Associated Lymphatic Vessels Upregulate PDL1 to Inhibit T-Cell Activation

**DOI:** 10.3389/fimmu.2017.00066

**Published:** 2017-02-03

**Authors:** Lothar C. Dieterich, Kristian Ikenberg, Timur Cetintas, Kübra Kapaklikaya, Cornelia Hutmacher, Michael Detmar

**Affiliations:** ^1^Institute of Pharmaceutical Sciences, Swiss Federal Institute of Technology (ETH) Zurich, Zurich, Switzerland; ^2^Department of Pathology and Molecular Pathology, University Hospital Zurich, Zurich, Switzerland

**Keywords:** peripheral tolerance, immune checkpoint, tumor vasculature, lymph node, PD1, abortive proliferation, T-cell exhaustion, tumor-induced immunosuppression

## Abstract

Tumor-associated lymphatic vessels (LVs) play multiple roles during tumor progression, including promotion of metastasis and regulation of antitumor immune responses by delivering antigen from the tumor bed to draining lymph nodes (LNs). Under steady-state conditions, LN resident lymphatic endothelial cells (LECs) have been found to maintain peripheral tolerance by directly inhibiting autoreactive T-cells. Similarly, tumor-associated lymphatic endothelium has been suggested to reduce antitumor T-cell responses, but the mechanisms that mediate this effect have not been clarified. Using two distinct experimental tumor models, we found that tumor-associated LVs gain expression of the T-cell inhibitory molecule PDL1, similar to LN resident LECs, whereas tumor-associated blood vessels downregulate PDL1. The observed lymphatic upregulation of PDL1 was likely due to IFN-g released by stromal cells in the tumor microenvironment. Furthermore, we found that blocking PDL1 results in increased T-cell stimulation by antigen-presenting LECs *in vitro*. Taken together, our data suggest that peripheral, tumor-associated lymphatic endothelium contributes to T-cell inhibition, by a mechanism similar to peripheral tolerance maintenance described for LN resident LECs. These findings may have clinical implications for cancer therapy, as lymphatic expression of PDL1 could represent a new biomarker to select patients for immunotherapy with PD1 or PDL1 inhibitors.

## Introduction

The lymphatic system comprises lymphatic capillaries and collecting vessels, as well as lymph nodes (LNs), and it exerts several essential functions in the body. Lymphatic vessels (LVs) take up interstitial fluid in peripheral tissues and transport it back to the blood circulation, thus maintaining basic tissue fluid homeostasis. At the same time, the lymphatic system provides a route for the recirculation of immune cells, such as memory T-cells, which constantly patrol the body, shuttling between the blood circulation and peripheral tissues. Similarly, dendritic cells and other antigen-presenting cells (APCs) use LVs to transport antigen taken up in the periphery to draining LNs, where they make contact with naive T- and B-lymphocytes to initiate adaptive immune responses. Consequently, the lymphatic system is closely connected to the immune system and the regulation of immune responses (1).

In pathological conditions, such as acute and chronic inflammation or cancer, peripheral LVs, and also draining LNs, undergo a dramatic expansion which is mediated by the enlargement of existing vessels as well as induction of *de novo* LV formation (lymphangiogenesis) (2–4). These effects are predominantly mediated by lymphangiogenic growth factors such as VEGF-C, produced at the site of inflammation or neoplastic growth. VEGF-C acts locally on nearby LVs, but may also be transported *via* the lymph to the draining LNs (5). Depending on the type of the inflammatory insult, the outcome of this expansion (and the concomitant increase in fluid drainage) may have beneficial or negative effects for the patient. For example, we and others have found that activation of LV expansion by administering VEGF-C decreases acute and chronic skin inflammation as well as rheumatoid arthritis (6–8), likely due to increased drainage of inflammatory factors and activated immune cells away from the site of inflammation. On the other hand, in cancer patients, an increased LV density in and around the tumor facilitates the lymphogenous spread of tumor cells and consequently correlates with LN metastasis and a poor prognosis (3, 4). At the same time, deficient lymphatic drainage in experimental tumor models reduces tumor inflammation and infiltration by immune effector cells, probably due to a lack of tumor-derived antigen reaching the local LNs which results in a state of “immunologic ignorance” of the tumor (9, 10).

Apart from these drainage-related effects, lymphatic endothelial cells (LECs) are also increasingly recognized as direct regulators of the immune system. LECs may act as non-professional APCs, expressing both MHC class I and class II molecules, which enable them to directly interact with T-cells and to modulate their activation status. This immune-regulatory function of LECs is particularly well studied in the case of LN resident LECs. Victor Engelhard and coworkers reported that LN LECs, but not LECs in peripheral LVs, express various self-antigens, including the melanocyte-specific antigen tyrosinase. Furthermore, LECs present peptides derived from these self-antigens on MHCI complexes to CD8+ T-cells and inhibit their activation in an antigen-dependent manner, thus eliminating autoreactive T-cells and maintaining peripheral tissue tolerance (11–13). LN LECs have also been found to take up free antigen from the lymph and to cross-present it to CD8+ T-cells, which may result in blunted T-cell responses to exogenous antigens (14). Taken together, the current data point to LN LECs being broadly inhibitory for CD8+ T-cells, both toward endogenous and exogenous antigens, at least under steady-state conditions. Whether LN LECs similarly interact with and inhibit CD4+ T-cells has remained somewhat controversial. On the one hand, LN LECs do express MHCII, but their ability to load it with antigen-derived peptides appears to be impaired due to a lack of H2-M expression (15). On the other hand, transfer of peptide-loaded MHCII complexes and/or antigen between LN LECs and other APCs, such as dendritic cells, has been reported, indicating that LN LECs may indeed play a role in the regulation of CD4+ T-cell responses (15, 16).

Various mechanisms how LN LECs control T-cells have been suggested, including a relative lack of co-stimulatory molecules and inhibition of T-cells *via* interaction of MHCII with LAG3 on the T-cell surface (11, 12, 15). In addition, LN LECs have been found to constitutively express the immune checkpoint molecule PDL1 (also called CD274 or B7H1), which inhibits T-cells *via* activation of the PD1 receptor, typically inducing a state of T-cell unresponsiveness termed “T-cell exhaustion” (17). However, in the case of peripheral tolerance induced by LN LECs *in vivo*, the effect on transferred autoreactive T-cells was reported to differ substantially from classical exhaustion, as those T-cells initially proliferated but subsequently became eliminated from the recipient mice (12), a process which has been termed “abortive proliferation.” In any case, the precise role of PDL1 expression in this process has not been entirely elucidated.

Steady-state PDL1 expression in LN LECs has been reported to be dependent on lymphotoxin signaling in the LN microenvironment (11). Additionally, PDL1 expression is inducible in various cell types, such as myeloid cells and endothelial cells (ECs), by inflammatory cytokines, particularly by IFN-g (18, 19). Therefore, PDL1 acts as a negative feedback regulator of Th1/CD8+ T-cell immune responses, which are characterized by high IFN-g release. Correspondingly, acute skin inflammation induced by repeated application of the contact sensitizer oxazolone, which triggers a Th1-biased immune response, resulted in a strong upregulation of PDL1 mRNA in isolated LECs (20). Similarly, PDL1 was upregulated in LECs upon antigen-specific interaction with CD8+ T-cells *in vitro* (14).

With regards to cancer, the role of LECs in regulating T-cell immunity is incompletely understood. Overexpression of VEGF-C in the B16F10 mouse melanoma model has been reported to decrease endogenous CD8+ T-cell responses against a model antigen (ovalbumin) and to turn these tumors refractory to adoptive T-cell transfer with OT-1 T-cells. Furthermore, these authors observed presentation of tumor antigen by peripheral and LN LECs, suggesting that LECs may contribute directly to the inhibition of T-cell-mediated antitumor immune responses (21). However, the mechanisms behind the T-cell inhibition by tumor-associated LECs have not been investigated so far.

We hypothesized that tumor-associated LECs might upregulate PDL1 in response to tumor-derived signals, and might thus contribute to the inhibition of tumor specific T-cells. Using two distinct syngeneic tumor models in different mouse strains, namely intradermal injection of VEGF-C overexpressing B16F10 melanomas and an orthotopic breast cancer model (4T1), we found that PDL1 is significantly upregulated in peripheral, tumor-associated LVs, presumably in response to IFN-g secreted by cells present in the tumor stroma. Using ovalbumin as model antigen, we provide direct evidence that PDL1 indeed reduces the stimulation of CD8+ T-cells by antigen-presenting LECs.

## Results

### PDL1 Is Upregulated in Tumor-Associated LVs

**Figure 1:**
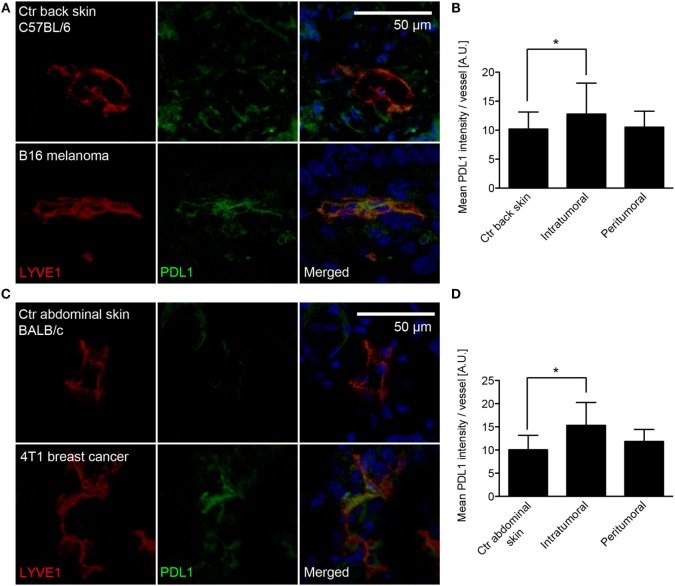
**PDL1 is expressed in tumor-associated lymphatic vessels (LVs)**. **(A)** Representative images of LVs (stained for LYVE-1, red) in control (Ctr) back skin (C57BL/6 background, top row) and B16F10-VEGFC melanoma (bottom row) co-stained for PDL1 (green). **(B)** Quantification of PDL1 staining intensity within the LYVE-1+ area of LVs in control back skin (*N* = 98 vessels from 10 individual mice), in the inner tumor mass of B16F10-VEGFC tumors (*N* = 17 vessels from six individual mice) and in the tumor periphery (*N* = 34 vessels from seven individual mice). **(C)** Similar to the B16F10-VEGFC model, PDL1 staining in LYVE-1-positive LVs was absent in the abdominal skin of BALB/c mice (top row) but was observed in 4T1 breast cancer-associated LVs (bottom row). **(D)** Quantification of PDL1 staining intensity within the LYVE-1+ area of LVs in control abdominal skin (*N* = 42 vessels from five individual mice), in the inner tumor mass of 4T1 tumors (*N* = 40 vessels from six individual mice), and in the tumor periphery (*N* = 35 vessels from five individual mice).

Previously, LVs in B16F10 melanomas overexpressing VEGF-C were reported to inhibit specific T-cell immunity (21), but the molecular mechanisms behind this inhibition have remained unclear. To investigate whether PDL1 is expressed in tumor-associated LVs, we implanted VEGF-C overexpressing B16F10 melanoma cells [B16F10-VEGFC (22)] intradermally into syngeneic C57BL/6 mice, and analyzed the expression of PDL1 by immunofluorescence staining of tumor sections 2 weeks later. Using LYVE-1 staining to identify LVs within the tumor mass and in the tumor periphery, we found increased PDL1 staining within the lymphatic endothelium of tumor-associated LVs, compared to LVs in the back skin of naive C57BL/6 mice (Figure 1A). In addition, diffuse PDL1 staining in a subset of tumor cells and in single, tumor-infiltrating cells could be observed. Image-based quantification of the PDL1 staining intensity in LYVE-1+ LVs confirmed a significant upregulation of the protein in intratumoral LVs but not in peritumoral LVs (Figure 1B).

To test whether lymphatic PDL1 expression is dependent on the tumor type, high expression of VEGF-C, or on the background mouse strain used, we next investigated PDL1 expression in a second, unrelated tumor model. 4T1 mammary carcinoma cells were implanted orthotopically in syngeneic hosts (Balb/c), and immunofluorescence stainings for PDL1 were performed 3 weeks later. Similar to what we found in melanomas, intratumoral LVs in 4T1 tumors also expressed PDL1, whereas PDL1 was not expressed by peritumoral LVs and LVs of the abdominal skin and the mammary fat pad of naive Balb/c mice (Figures 1C,D).

**Figure 2:**
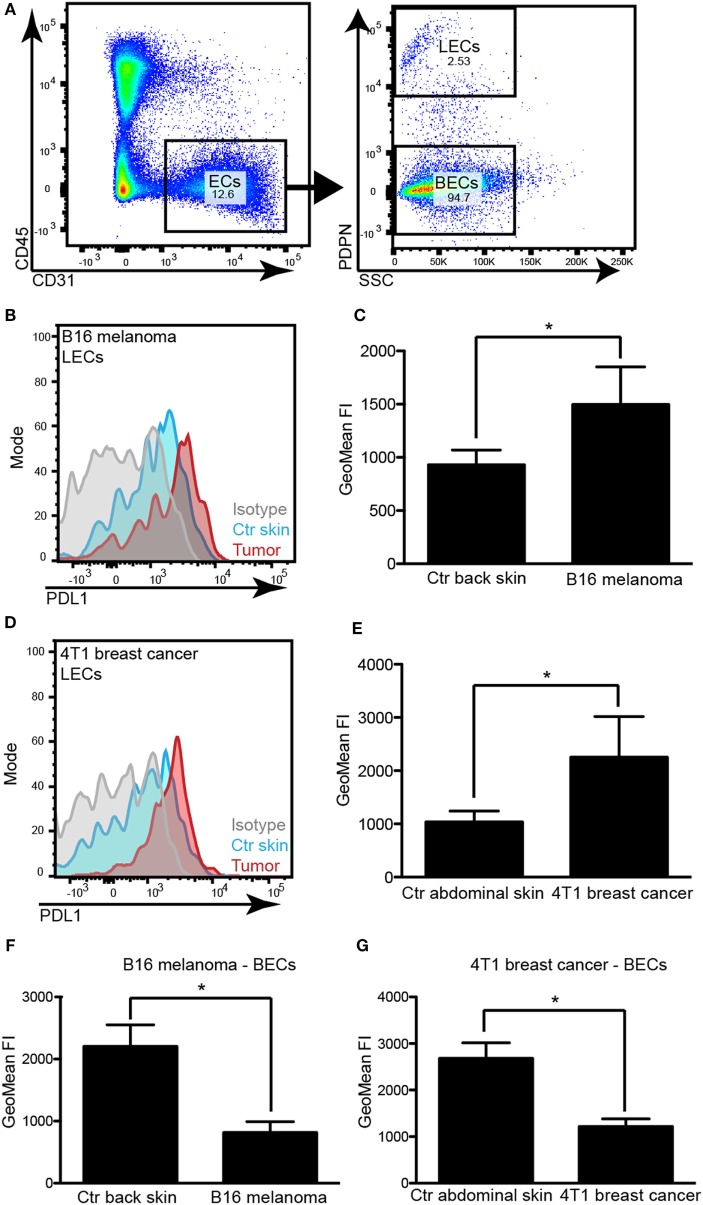
**PDL1 is upregulated in tumor-associated lymphatic vessels but down-regulated in tumor-associated blood vessels**. **(A)** Example FACS plots to illustrate the gating strategy used to identify lymphatic endothelial cells (LECs) and blood vascular endothelial cells (BECs) in skin samples and tumors. Left panel: total endothelial cells (ECs) were identified as CD31+CD45− cells in a control skin sample (pre-gated for living singlets). Right panel: LECs were differentiated from BECs by staining for podoplanin (PDPN). **(B)** Example histogram of PDL1 staining intensity measured by FACS in LECs of control (Ctr) back skin and B16F10-VEGFC tumors. **(C)** Quantification of staining intensity in control (Ctr) back skin and B16F10-VEGFC-associated LECs (*N* = 5 mice/group). **(D)** Example histogram of PDL1 staining intensity measured by FACS in LECs of control abdominal skin and 4T1 tumors. **(E)** Quantification of staining intensity in control abdominal skin and 4T1-associated LECs (*N* = 4 mice/group; one out of two experiments with similar results is shown). **(F,G)** Quantification of PDL1 staining intensity in BECs of B16F10-VEGFC tumors [**(F)**, *N* = 5 mice/group) and of 4T1 tumors [**(G)**, *N* = 4 mice/group; one out of two experiments with similar results is shown].

As PDL1 might be expressed by various cell types, including immune cells which sometimes reside in very close proximity to LVs and may thus confound the microscopic analysis, we next performed FACS analyses of primary B16F10-VEGFC and 4T1 tumors compared to the corresponding control tissues. Antibodies against CD45, CD31, and podoplanin (PDPN) were used to differentiate between immune cells, blood vascular endothelial cells (BECs), and LECs (Figure 2A). Analysis of the fluorescence intensity confirmed that in both tumor models, tumor-associated LECs expressed higher levels of PDL1 than LECs in normal skin (Figures 2B–E). It is of interest that PDL1 expression on BECs showed the opposite behavior, with a significant reduction in PDL1 expression in tumor-associated BECs compared to normal skin BECs (Figures 2F,G), indicating that BECs react very differently to stimuli derived from the tumor microenvironment. To investigate whether PDL1 expression by LVs might be induced only in very late stages of tumor growth, and might thus not be relevant for the inhibition of T-cell responses, which are likely triggered during the early growth phase of tumors, we next analyzed lymphatic PDL1 expression already 8 days after 4T1 implantation. Similar to our observation at the 3-week time point, we found PDL1 induced in LECs and reduced in BECs (Figure S1 in Supplementary Material).

### PDL1 Expression in LN Stromal Cells Is Not Affected by the Presence of an Upstream Tumor

**Figure 3:**
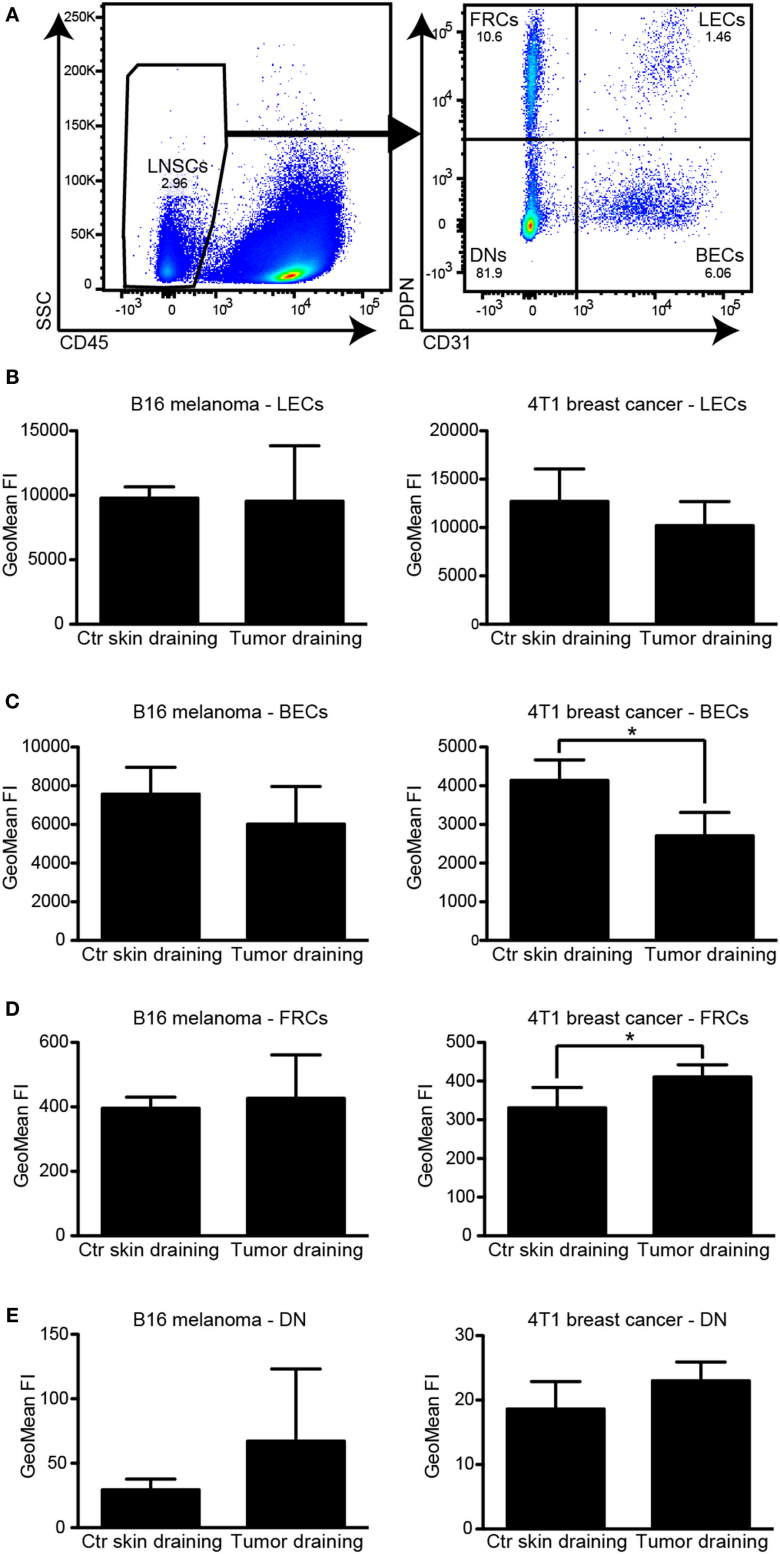
**PDL1 is constitutively expressed in lymph node (LN) lymphatic endothelial cells (LECs) and is not affected by the presence of an upstream tumor**. **(A)** Example FACS plots of a LN (pre-gated for living singlets). CD45− LN stromal cells (left, LNSC) were separated into podoplanin (PDPN)+/CD31− follicular reticular cells (FRCs), PDPN+/CD31+ LECs, PDPN−/CD31+ BECs, and PDPN−/CD31− “double negative” (DN) cells (right panel). **(B–E)** Quantification of PDL1 staining intensity by FACS in LN LECs **(B)**, BECs **(C)**, FRCs **(D)**, and DNs **(E)** in B16F10-VEGFC draining inguinal LNs (left panels, *N* = 5 mice/group) and 4T1 draining inguinal LNs (right panels, *N* = 4 mice/group; one out of two experiments with similar results is shown), compared to control (Ctr) LNs in naive mice. LECs expressed the highest levels of PDL1. Only minor changes in PDL1 expression levels of BECs and FRCs in 4T1 draining LNs were observed.

Lymph node LECs have previously been reported to express PDL1 under steady-state conditions, depending on lymphotoxin signaling (11). Cytokines and growth factors drained from an upstream inflammatory site or a tumor mediate expansion of the lymphatic vasculature of the draining LN [reviewed in Ref. (2)], but their effect on the PDL1 expression by LN LECs has not been investigated. We therefore analyzed the PDL1 expression in the major LN stromal cell subsets [CD31+/podoplanin+ LECs, CD31+/podoplanin− BECs, CD31−/podoplanin+ follicular reticular cells (FRCs), and CD31−/podoplanin− double negative (DN) cells; Figure 3A] in tumor draining inguinal LNs and in inguinal LNs of naive mice. In agreement with previous reports (12), we found that LECs expressed the highest PDL1 levels among those cell types. The surface levels of PDL1 did not change significantly in response to the presence of an upstream tumor (Figure 3B). BECs also expressed considerable amounts of PDL1 on their cell surface. In line with our observations in primary tumors, PDL1 expression on LN BECs was reduced in tumor bearing mice, especially in the 4T1 breast cancer model (Figure 3C). PDL1 expression in FRCs and in DN cells was generally very low and was only slightly increased by the presence of an upstream tumor (Figures 3D,E). Taken together, these data indicate that LN LEC expression of PDL1 is constitutively high and is not affected by cytokines or other factors drained from upstream tumors.

### PDL1 Expression in LECs Is Regulated by IFN-g

**Figure 4:**
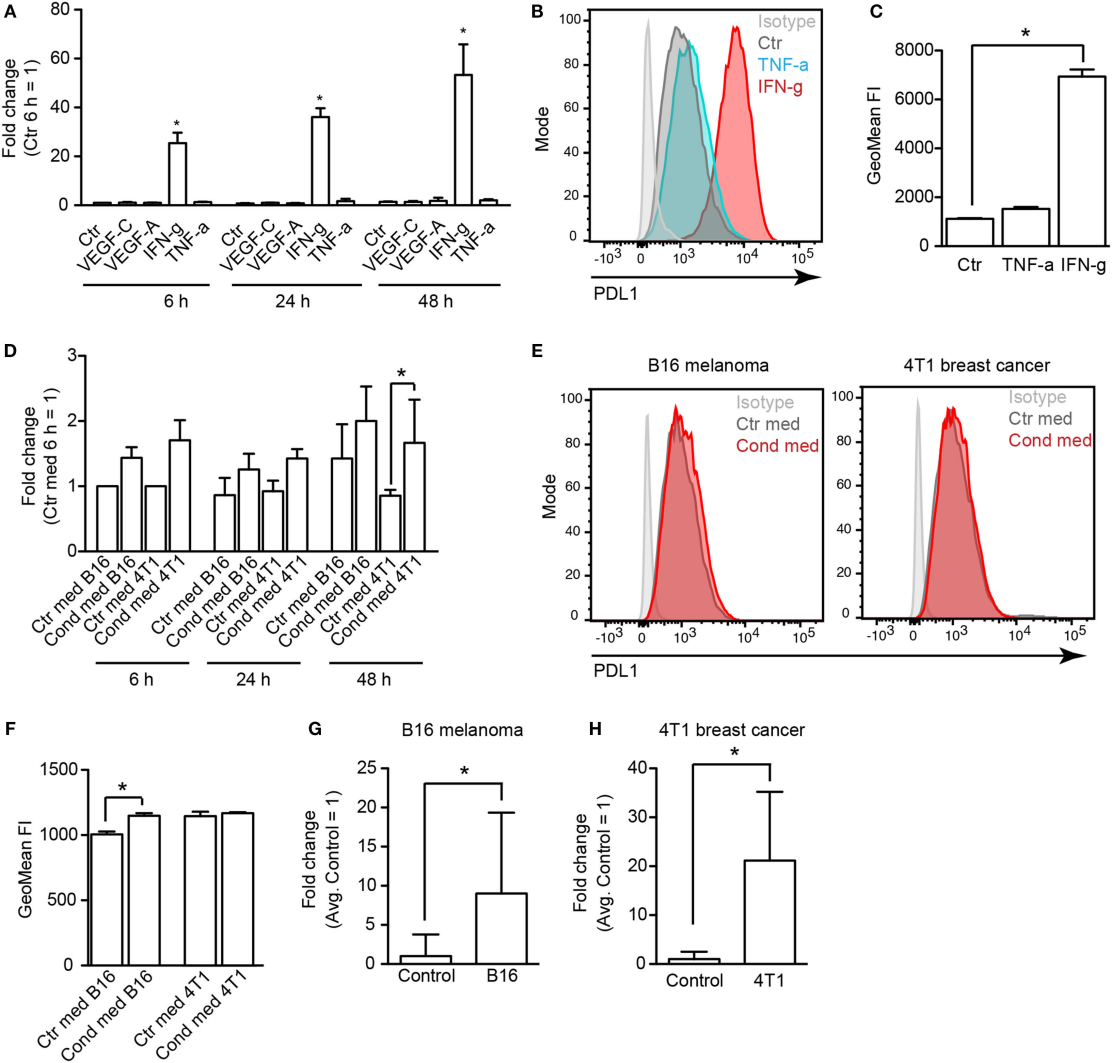
**Lymphatic endothelial cells (LECs) upregulate PDL1 in response to IFN-g**. **(A)** Immortalized mouse LECs (imLECs) were treated with VEGF-A (20 ng/ml), VEGF-C (200 ng/ml), TNF-a (40 ng/ml), or IFN-g (100 ng/ml). Expression of PDL1 was assessed by qPCR after 6, 24, and 48 h. Incubation with IFN-g resulted in a significant upregulation of PDL1 mRNA (pooled data of three individual experiments are shown). **(B)** Example histogram of surface PDL1 expression assessed by FACS in imLECs treated with TNF-a or IFN-g or not (Ctr). **(C)** Quantification of surface PDL1 in untreated (Ctr), TNF-a treated, and IFN-g treated imLECs (*N* = 3). **(D)** qPCR showing PDL1 expression upon imLEC treatment with tumor cell conditioned media (Cond med) derived from B16F10-VEGFC or 4T1 cells, compared to control media (Ctr med) (pooled data of three individual experiments are shown). **(E)** Example histograms of surface PDL1 expression assessed by FACS in imLECs treated with B16F10-VEGFC (left) or 4T1 cell conditioned medium (right). **(F)** Quantification of surface PDL1 expression in imLECs treated with control (Ctr med) or tumor cell conditioned media (Cond med) (*N* = 3). **(G,H)** qPCR data showing IFN-g expression in B16F10-VEGFC **(G)** and 4T1 tumor tissue **(H)** compared to control tissue (back skin resp. abdominal skin) (*N* = 7–9 for B16F10-VEGFC and 4–7 for 4T1).

To elucidate how PDL1 expression is regulated in tumor-associated LECs, we treated immortalized mouse LECs (imLECs) with several (lymph-) angiogenic growth factors (VEGF-A, VEGF-C) and inflammatory cytokines (IFN-g, TNF-a), all of which are commonly expressed in the microenvironment of various tumors. Using qPCR, we found a strong upregulation of PDL1 mRNA expression in imLECs treated with IFN-g (up to 40-fold after 24 h). TNF-a had only very minor effects on PDL1 expression (up to twofold induction), whereas VEGF-A and VEGF-C had no effect at all (Figure 4A). IFN-g mediated induction of PDL1 expression was also reflected at the protein level: using FACS analysis, we found that imLECs constitutively express PDL1 on their surface and dramatically upregulated it upon treatment with IFN-g for 24 h (Figures 4B,C). To test whether tumor cells can directly induce PDL1 expression in LECs *via* secretion of one or several soluble factors (e.g., IFN-g), we treated imLECs with conditioned media from B16F10-VEGFC and 4T1 cells. Using qPCR and FACS, we only detected a minor induction of PDL1 by tumor cell conditioned media (Figures 4D–F). In line with this, IFN-g expression was not detectable in cultured B16F10-VEGFC cells and in 4T1 cells (data not shown), whereas IFN-g expression in total tumor tissue was readily detectable and was increased compared to the skin of corresponding naive mice in both tumor models (Figures 4G,H). Taken together, these findings suggest that cells present in the tumor stroma, for example infiltrating immune cells, are responsible for the induction of PDL1 expression in tumor-associated LVs *via* secretion of IFN-g.

### T-Cells Physically Interact with Tumor-Associated LVs *In Vivo*

**Figure 5:**
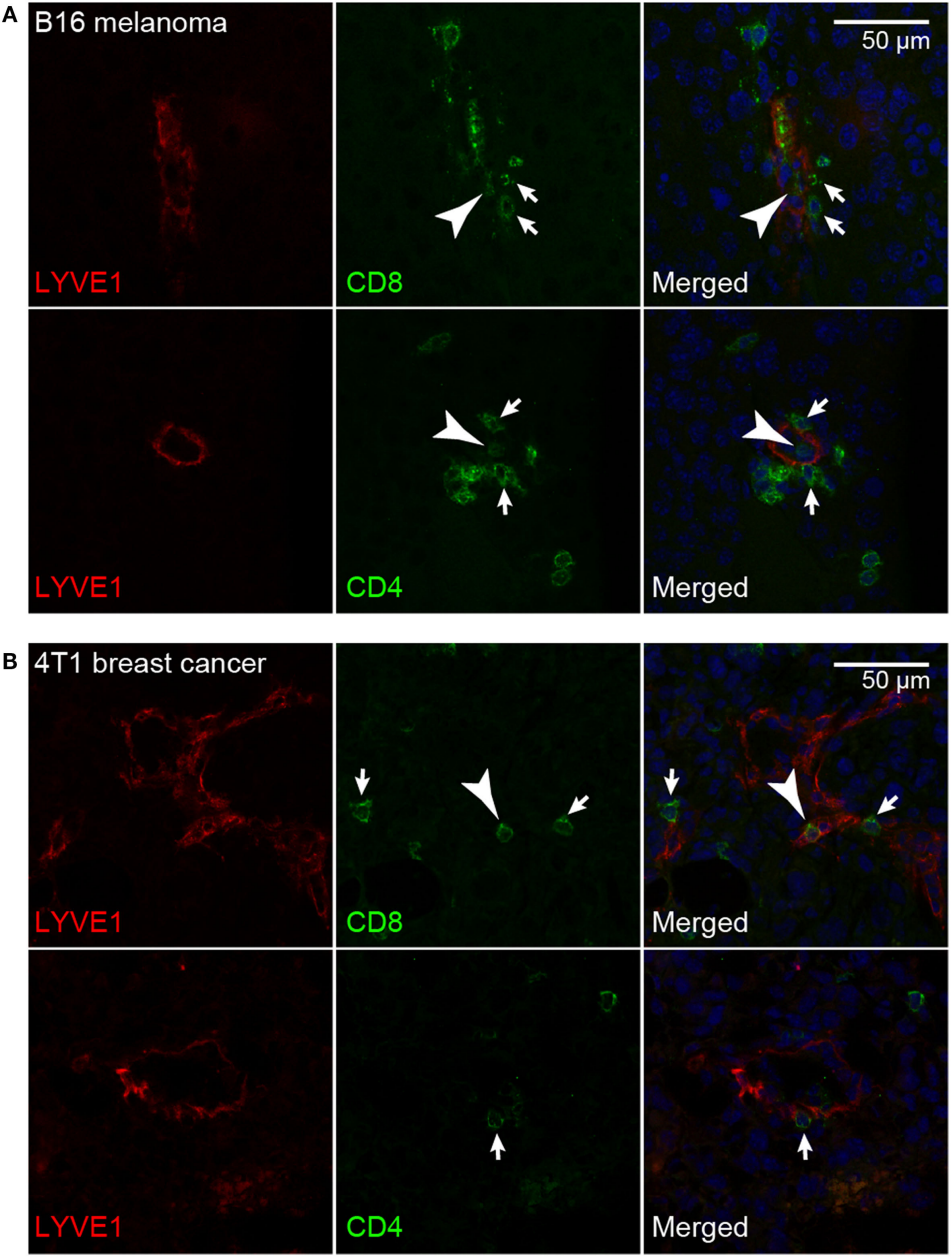
**T-cells interact with tumor-associated lymphatic vessels**. Representative images of CD8+ and CD4+ T-cells (green) interacting with LYVE-1+ lymphatic endothelial cells (red) in B16F10-VEGFC melanoma **(A)** and 4T1 breast cancer **(B)**. T-cells interacting with the abluminal (arrows) and the luminal (arrowheads) surface of the lymphatic endothelium were observed in both tumor models.

Previously, it has been reported that in VEGF-C overexpressing B16F10 melanomas, adoptively transferred T-cells were often clustering around tumor-associated LVs, indicating a physical interaction between T-cells and LECs (21). Using double immunofluorescence stainings for the lymphatic marker LYVE-1 and for CD4 or CD8 to identify helper T-cells and cytotoxic T-cells in B16F10-VEGFC tumors, we found that endogenous T-cells, although relatively few in number, occasionally interacted with LECs, both at the luminal and the abluminal side of the endothelium (Figure 5A). Very similar results were obtained when we analyzed 4T1 breast cancer tissue (Figure 5B). Given that tumor-associated LVs express elevated levels of PDL1, this finding suggests that tumor-infiltrating T-cells may receive inhibitory signals from LECs either while still residing in the tumor stroma, or upon exiting it *via* the lymphatic system in route toward the draining LNs.

### PDL1 Inhibits Antigen-Dependent Activation of T-Cells by LECs

**Figure 6:**
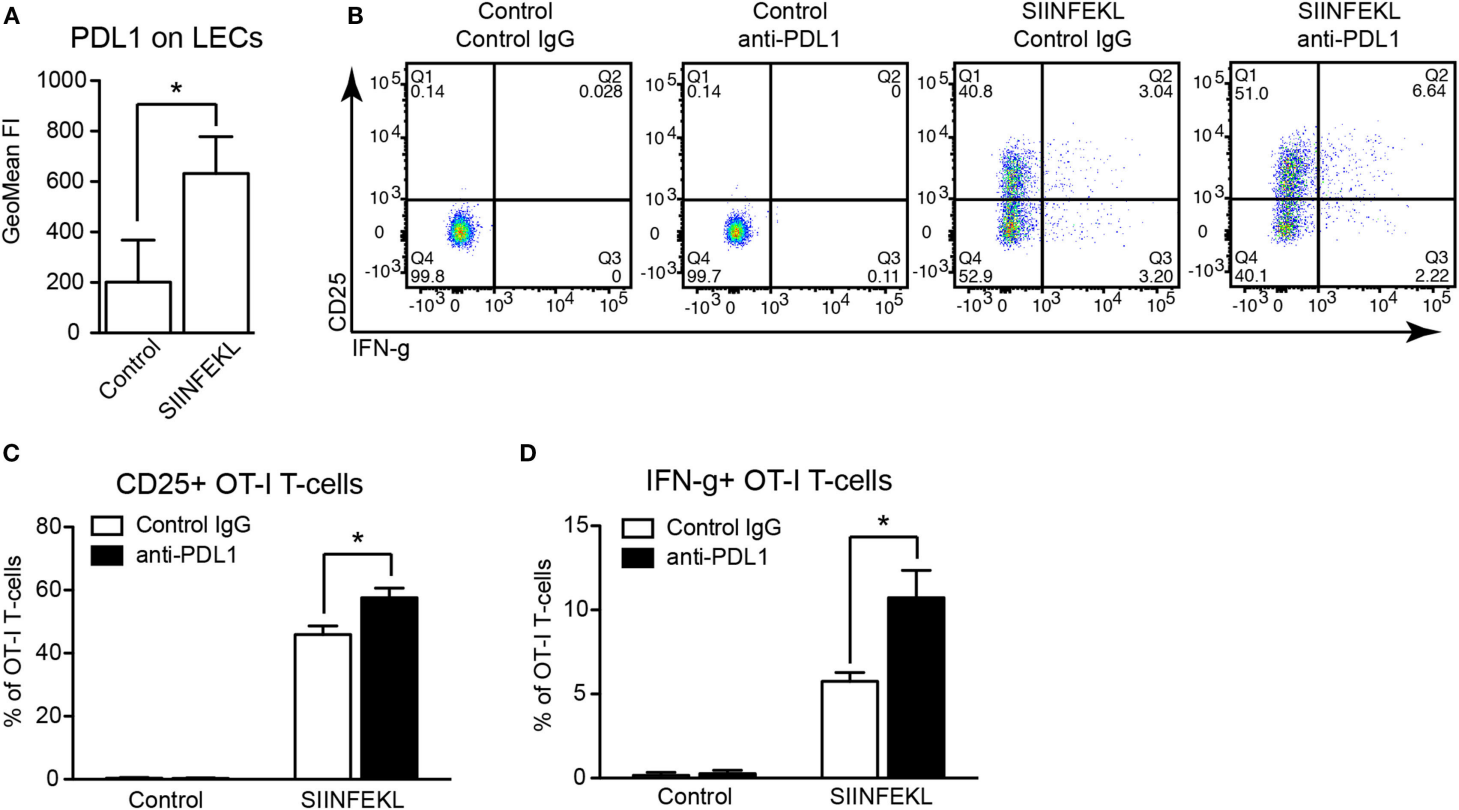
**PDL1 blockade increases antigen-specific stimulation of CD8+ T-cells by lymphatic endothelial cells (LECs)**. **(A)** PDL1 surface expression on immortalized mouse LECs (imLECs) cocultured with OT-1 CD8+ T-cells and pulsed with SIINFEKL peptide or not (Control) was determined by FACS (*N* = 3). **(B)** Representative FACS plots of CD25 and IFN-g expression by OT-1 CD8+ T-cells (pre-gated for living singlets) after coculture with control or SIINFEKL-pulsed imLECs, in the presence of PDL1-blocking antibodies or control IgG. **(C,D)** Quantification of CD25+ OT-1 cells **(C)** and IFN-g+ OT-1 cells **(D)** by FACS gated as in panel **(B)** (*N* = 3).

Previously, it has been reported that presentation of the ovalbumin-derived, MHCI-restricted peptide SIINFEKL by cultured imLECs to OT-1 CD8+ T-cells reduces their activation as compared to peptide-presenting dendritic cells, with a blunted upregulation of CD25 and reduced expression of IFN-g by OT-1 cells (14). Whereas upregulation of PDL1 by peptide-presenting imLECs was observed, its role in imLEC–OT-1 interaction was not investigated (14). We hypothesized that PDL1 expressed (and upregulated) by antigen-presenting imLECs might at least in part be responsible for the reduced OT-1 stimulation. Using the same *in vitro* model system, we confirmed that imLECs upregulate surface PDL1 upon SIINFEKL peptide presentation to OT-1 T-cells, whereas PDL1 was not upregulated in absence of a specific antigen (Figure 6A). Next, we used a PDL1-blocking antibody to determine the role of PDL1 during imLEC-mediated OT-1 activation. FACS analyses revealed that blockade of PDL1 increased the expression of CD25 and IFN-g in OT-1 cells upon coculture with SIINFEKL-presenting imLECs (Figures 6B–D). Increased activation of OT-1 cells after PDL1 blockade also resulted in an elevated capacity to kill ovalbumin-expressing B16F10 tumor cells *in vitro* (Figure S2A in Supplementary Material). Interestingly, we also observed a strong upregulation of PDL1 by antigen-stimulated OT-1 T-cells themselves (Figure S2B in Supplementary Material), likely due to a paracrine effect of T-cell secreted IFN-g. Thus, it is possible that the increased activation of OT-1 T-cells by antigen-presenting imLECs after PDL1 blockade is partly due to inhibition of OT-1 expressed PDL1, in addition to imLEC expressed PDL1. Therefore, we performed additional experiments in which PDL1 was specifically blocked on the surface of imLECs before coculture with OT-1 cells. Also in this setting, we found a potentiating effect of PDL1 blockade on OT-1 stimulation, resulting in elevated CD25 expression (Figure S2C in Supplementary Material), strongly suggesting that LEC expressed PDL1 indeed contributes to LEC-mediated T-cell inhibition.

## Discussion

Adaptive immune responses require the cooperation between cells of the immune system and the lymphatic vasculature. LNs are the principal site where naive T- and B-lymphocytes encounter antigen and where the decision is taken whether these lymphocytes become primed to proliferate and develop into effector cells, or whether they enter anergy, or even become eliminated (peripheral tolerance). Foreign and self-antigens are transported to the LNs *via* the lymphatic system, either as “cargo” transported by professional APCs, or as free molecules or complexes carried with the lymph flow. Once arrived at the LN, antigens are transferred into the T- and B-cell zones, either by the LN conduit system, or by LN resident or migratory APCs.

With regards to tumor immunology, some priming of naive T-cells has been reported to occur within the tumor microenvironment [reviewed in Ref. (23)]; yet, draining LNs are still the primary site for the mounting of adaptive immune responses. Consequently, using transgenic mice, a lack of or disturbed lymphatic draining from the tumor site resulted in reduced immune activation, immune cell infiltration into the tumor, and cytokine release (9, 10), indicating that antigen transport by LVs is required for efficient antitumor immune responses. On the other hand, induction of lymphangiogenesis by forced expression of VEGF-C resulted in a blunted T-cell response in the B16F10 melanoma model (21), suggesting that the lymphatic endothelium, within the tumor and/or the draining LNs, has a direct, T-cell inhibitory function. Interestingly, tumor-associated LECs have been reported to present tumor-derived antigen (ovalbumin) on MHCI (21). Using the same tumor model (VEGF-C expressing B16F10 melanoma), we demonstrate here that tumor-associated LECs upregulate the T-cell inhibitory immune checkpoint molecule PDL1. Our data also show that this is neither dependent on the tumor model (B16F10) nor on the genetic background (C57BL6) or the experimental overexpression of VEGF-C, as PDL1 was also upregulated in a completely unrelated tumor model, the 4T1 breast cancer model in the Balb/c background.

Presentation of antigen on MHCI in combination with PDL1 expression has been found to be a specific feature of LN LECs (11, 12). This phenomenon has been linked to the maintenance of peripheral immune tolerance, as LN LECs express and present various self-antigens, at least under steady-state conditions, reminiscent of the thymic epithelium which is responsible for central tolerance (13). The fact that PDL1 is upregulated on tumor-associated LECs as found here, as well as in acutely inflamed LECs (20), suggests that under pathological conditions, a very similar T-cell inhibitory mechanism is activated in peripheral LVs as well. Of note, we and others have shown that induction of lymphangiogenesis in inflammatory conditions ameliorates inflammation and promotes resolution (6–8). It is tempting to speculate that direct inhibition of T-cells, in addition to increased lymph drainage, may contribute to this effect. In the tumor context, multiple pathways and factors have been identified that inhibit the activity of tumor-infiltrating lymphocytes, resulting in T-cell anergy or exhaustion. Peripheral LECs presenting tumor-derived antigens with concomitant PDL1 upregulation by tumor-associated LECs may to some extent contribute to overall T-cell inhibition in the tumor microenvironment, which is consistent with the observations made before (21). On the other hand, it is intriguing that PDL1 expression on tumor-associated LVs may particularly affect antigen-experienced T-cells, including memory T-cells, which have the capacity to recirculate from the tumor site to the circulation *via* the lymphatic system. Therefore, further studies are highly warranted to elucidate whether tumor-associated LECs particularly inhibit the development of memory responses. Inhibition of recirculating T-cells entering LVs may also have a negative impact on the priming of additional naive T-cells in downstream LNs.

PDL1 is an IFN-g target gene in microvascular ECs (18), and our data reveal that IFN-g treatment induces PDL1 expression in cultured LECs as well. As we found IFN-g to be expressed in the tumor microenvironment of both tumor models that we studied, we suggest that the observed PDL1 upregulation in tumor-associated LECs is in fact mediated by IFN-g. Consistently, we found no major PDL1 induction by tumor cell conditioned media, which did not contain significant amounts of this cytokine. Therefore, the regulation of PDL1 in LVs in the periphery differs from that in the LNs, where it was reported to be dependent on lymphotoxin, a specific activator of non-canonical NF-kB signaling (11). As we found no significant effect of TNF-a on PDL1 expression, at least *in vitro*, it is likely that only the non-canonical, but not the canonical, NF-kB pathway regulates PDL1 expression in LN LECs. In line with this concept, the PDL1 expression in LN LECs was not affected by the presence of a tumor and thus, by tumor-derived factors drained to the LNs.

Surprisingly, we observed the opposite effect on tumor-associated blood vessels. In contrast to LECs, we found that peripheral BECs in the skin express PDL1 under steady-state conditions, but that they downregulate it in presence of a tumor. Possibly, PDL1 upregulation by IFN-g is blocked in tumor-associated BECs due to high expression of VEGF-A and the induction of angiogenesis, which may interfere with inflammatory activation of BECs (24, 25). Functionally, PDL1 expression on BECs has been reported to inhibit autoreactive CD8+ T-cells in a myocarditis model (26), indicating that reduced PDL1 expression in tumor-associated BECs might facilitate infiltration of activated effector T-cells into the tumor stroma. However, further studies are needed to test this hypothesis and to investigate whether the PDL1 expression level on tumor BECs is higher in tumor models that show no T-cell infiltration at all.

Despite the findings that PDL1 is constitutively expressed by LN LECs (11, 12) and that it is upregulated on tumor-associated LECs as identified here, its precise role in LEC-mediated T-cell inhibition is not entirely clear. Treating naive mice with PDL1-blocking antibodies resulted in the development of autoimmune vitiligo after transfer of T-cells specific for the melanocyte-specific antigen tyrosinase (12). Using chimeric mice carrying PDL1−/− bone marrow, it was furthermore reported that the inhibition of autoreactive T-cells was mediated by a PDL1+, radioresistant stromal cell type, consistent with LN LECs being responsible for the maintenance of peripheral tolerance toward tyrosinase in this model (12). Similarly, cultured LECs presenting the ovalbumin-derived SIINFEKL peptide to OT-1 CD8+ T-cells have been described to upregulate PDL1 and to activate OT-1 cells much less effectively than dendritic cells, resulting in reduced expression of the activation marker CD25 and production of IFN-g (14). Nonetheless, no direct evidence that PDL1 expression by LECs is indeed required and/or sufficient for T-cell inhibition has been published so far. Using the same *in vitro* system of cultured LECs presenting the SIINFEKL peptide to OT-1 cells, we reveal here that inhibition of PDL1 with a blocking antibody indeed increases CD25 and IFN-g expression by OT-1 cells, and harnesses them for killing of ovalbumin-expressing tumor cells. As OT-1 cells themselves strongly upregulate PDL1 in this setting (indicating that PDL1 expression might serve as a sensitive marker of T-cell activation), we also pre-blocked PDL1 specifically on the LEC surface before coculturing them with OT-1 cells. This way of PDL1 blockade is conceivably less efficient than adding the blocking antibodies throughout the coculture period, due to additional PDL1 upregulation by the imLECs during this time period. Furthermore, dynamic antibody binding and dissociation may still result in some PDL1 blockade on the OT-1 T-cells during the coculture. Nonetheless, our observation that the activation of OT-1 cells was increased even in this setting further supports the notion that LEC expressed PDL1 at least partially dampens the activation of T-cells by antigen-presenting LECs. However, based on our data, an additional role of T-cell expressed PDL1 acting as a negative feedback regulator of T-cell activation cannot be entirely excluded at this moment.

Clinically, the PDL1 expression in cancer has lately received considerable attention due to the development of highly potent PD1-blocking antibodies (nivolumab and pembrolizumab) as well as PDL1-blocking antibodies (e.g., atezolizumab and durvalumab) which show dramatic improvements of outcome in melanoma patients or are in advanced clinical studies, respectively (27). However, only a subset of patients profits from the treatment, and the search for predictive biomarkers to reliably identify those patients is ongoing. PDL1 expression on cancer cells is currently regarded as such a biomarker and has recently been approved by the US Food and Drug Administration as a companion or complementary diagnostic test for certain specific tumor entities. However, there are difficulties and inconsistencies in the clinical protocols to determine the rate of PDL1+ cancer cells in biopsy material as there is no fully standardized detection antibody, definition of cutoffs for the evaluation of the stainings, tissue preparation, and processing protocols available yet. Furthermore, it is debatable whether the currently widely used detection in cancer cells itself is the optimal predictive marker (28). In this regard, it is interesting that a recent study performed in patients with multiple different tumor types found that PDL1 expression in stromal cells, including tumor-infiltrating leukocytes, was superior in predicting patients who would benefit from treatment with a new PDL1-blocking antibody currently under development (29). Furthermore, there is growing evidence that at least for some malignancies, patients treated with anti-PD1 antibodies have a survival benefit independent of the expression of its ligand PDL1 on tumor cells (30). Thus, it is likely that additional (stromal) cell types are involved in the inhibition of antitumor T-cell responses *via* the PDL1-PD1 axis. In the light of our findings presented here, assessment of the vascular PDL1 expression may yield additional predictive accuracy and may further improve the selection of patients to undergo treatment with PD1 or PDL1 inhibitors in the future.

## Materials and Methods

### Cell Lines

B16F10 cells expressing luciferase and human VEGF-C have been generated and described previously (22). Cells were cultured in DMEM containing Glutamax, pyruvate, 10% FBS, and penicillin/streptomycin (all from Gibco/Thermo Fisher). G418 (1.5 mg/ml, Roche) was added to the culture medium to ensure stable expression of the VEGF-C transgene. 4T1 mammary carcinoma cells expressing luciferase (Caliper Life Sciences) were maintained in DMEM supplemented with l-glutamine, 10% FBS, and penicillin/streptomycin under standard culture conditions (37°C, 5% CO_2_). ImLECs isolated from *H-2Kb-tsA58* (Immorto) mice have been described previously (20) and were maintained on collagen type I (Advanced Biomatrix)/fibronectin (Millipore) coated dishes (10 µg/ml each) in Ham’s F12/DMEM supplemented with 20% FBS, 56 µg/ml heparin (Sigma), 10 µg/ml EC growth supplement (Abd Serotec/BioRad), penicillin/streptomycin, and 1 U/ml recombinant mouse IFN-g (Peprotech) at 33°C, 5% CO_2_. Before experiments, cells were cultured at 37°C without IFN-g for at least 72 h, which leads to a reduction of the polyoma T antigen in these cells.

### Mice and Tumor Models

C57BL/6 and Balb/c wild-type mice were obtained from Janvier. OT-1 transgenic mice were kindly provided by Dr. Roman Spörri and Dr. Annette Oxenius, ETH Zurich. All mice were bred in house under SOPF conditions. For the B16F10-VEGFC melanoma model, C57BL/6 mice were depilated on the back and 2 × 10^5^ tumor cells suspendend in 20 µl PBS were injected intradermally into the flank. For the 4T1 breast cancer model, 1 × 10^5^ tumor cells suspended in 50 µl PBS were injected into the fourth mammary fat pad of Balb/c mice. The primary tumor growth was monitored for 2 weeks (B16F10-VEGFC) or 3 weeks (4T1) before tissues were prepared for analysis as described below. In case of the 4T1 model, some FACS analyses were already performed at day 8 after implantation. All tumor studies were performed in agreement with the regulations of the local ethical board (Kantonales Veterinäramt Zürich, license 12/15).

### Immunofluorescence Staining and Image Analysis

Tumors were dissected, embedded in OCT compound, snap frozen, and stored at −80°C until preparation of cryosections (7 µm). For stainings, sections were air-dried, fixed in ice-cold acetone and 80% methanol, rehydrated in PBS, and subsequently blocked in PBS + 0.2% BSA, 5% donkey serum, 0.3% Triton-X100, and 0.05% NaN_3_ (blocking solution). Primary antibodies [rabbit anti-LYVE-1 (1:600, Angiobio), rat anti-PDL1 (2 µg/ml, clone 10F.9G2, Biolegend), rat anti-CD4 (5 µg/ml, clone H129.19, BD), and rat anti-CD8 (5 µg/ml, clone 53.6-7, BD)] suspended in blocking solution were incubated at room temperature for 2 h or at 4°C over night, followed by extensive washing and incubation with Alexa488 or Alexa594-conjugated secondary antibodies (donkey anti-rat, donkey anti-rabbit, 10 µg/ml, Life Technologies/Thermo Fisher) together with Hoechst33342 (2 µg/ml, Sigma) for nuclear counterstaining. Finally, slides were washed extensively again and mounted using Mowiol.

Images were taken on a Zeiss Axioskop 2 mot plus with a 10× or a 20× objective, or a Zeiss LSM780 inverted confocal microscope at 20×. Image analysis was performed using ImageJ (NIH). To determine the staining intensity of PDL1 in LVs, LYVE-1+ vessels were selected by thresholding, using size exclusion to exclude single LYVE-1+ macrophages. Subsequently, the average PDL1 staining intensity within each LV was measured.

### FACS Analysis of Tumor Tissue and LNs

For FACS analysis of tumors and control tissues (back skin and abdominal skin, respectively), the tissue was dissected, minced, and digested in a collagenase solution [5 mg/ml Collagenase II (Sigma), 40 µg/ml DNaseI (Roche)] for 30 min at 37°C. The digested tissue was passed through a cell strainer before erythrocyte lysis with PharmLyse (BD). After washing and a second filtration step, the cell suspension was labeled with fluorescently tagged antibodies [hamster anti-podoplanin-PE (1:400, clone 8.1.1, eBioscience), rat anti-CD31-APC (1:300, clone MEC13.3, BD), rat anti-CD45-APC/Cy7 (1:200, clone 30-F11, Biolegend), and rat anti-PDL1-PE/Cy7 (1:200, clone 10F.9G2, Biolegend)]. 7AAD (Biolegend) was used for life/dead discrimination. Inguinal LNs were processed essentially as described before (31). In brief, the capsule of dissected LNs was ruptured and the tissue was digested with 1 mg/ml Collagenase IV (Gibco)/40 µg/ml DNaseI for 20 min at 37°C to release the majority of the immune cells. The remaining stromal fragments were washed twice, digested with 3.5 mg/ml Collagenase IV/40 µg/ml DNaseI for 15 min at 37°C, and disaggregated by pipetting in presence of 0.5 mM EDTA. After filtration, the stromal cell enriched cell suspension was stained as described above. Data were acquired on a FACS CANTO (BD) and analyzed using FlowJo (Treestar Inc.).

### LEC Stimulation *In Vitro*

imLECs were starved over night in Ham’s F12/DMEM + 1% FBS and subsequently stimulated with VEGF-A (20 ng/ml, Cell Sciences), VEGF-C (200 ng/ml, R&D), IFN-g (100 ng/ml, Peprotech), or TNF-a (40 ng/ml, Peprotech). Tumor cell conditioned media were prepared by culturing 1 × 10^7^ B16F10-VEGFC or 4T1 cells in medium supplemented with 1% FBS for 72 h. The conditioned media were centrifuged, filtered through a 0.45 µm filter, and stored at −80°C until use. ImLECs were stimulated with 50% conditioned media in Ham’s F12/DMEM + 1% FBS. DMEM with Glutamax and pyruvate, or DMEM with l-glutamine (both with 1% FBS) served as control media. For FACS analysis, LECs were washed with PBS and trypsinized with 0.01% trypsin/EDTA. Cells were labeled with primary antibody (rat anti-PDL1, 2 µg/ml, clone 10F.9G2, Biolegend), washed and labeled with a secondary antibody (donkey anti-rat-Alexa488, 10 µg/ml, Life Technologies/Thermo Fisher). 7AAD was used for life/dead discrimination. Data were acquired on a FACS CANTO and analyzed using FlowJo.

### RNA Extraction and qPCR

RNA from stimulated LECs was extracted at the indicated time points using the Nucleospin RNA kit (Macherey-Nagel) according to the manufacturer’s instructions. Total tumor and control tissue RNA was extracted from cryosections using the RNeasy Plus Micro kit (Qiagen). All RNA was reverse transcribed using the High Capacity cDNA kit (Applied Biosystems/Thermo Fisher). qPCR analyses were performed on a 7900HT FAST instrument (Applied Biosystems/Thermo Fisher) in triplicate using SYBRGreen (Roche). RPLP0 was used as internal reference gene. Relative expression (RE) calculated according to the formula ${\text{RE}}_{\text{geneX}}=2^{-\left({\text{Ct}}_{\text{geneX}}-{\text{Ct}}_{\text{RPLP0}}\right)}$ and was expressed as fold change normalized to the control condition. The primer sequences used for qPCR were RPLP0 fwd: AGATTCGGGATATGCTGTTGG, rev: TCGGGTCCTAGACCAGTGTTC; PDL1 fwd: ACAAGCGAATCACGCTGAAAG, rev: GGCCTGACATATTAGTTCATGCT; and IFNG fwd: ACACTGCATCTTGGCTTTGC, rev: CTGGCTCTGCAGGATTTTCA.

### OT-1 T-Cell Activation and Tumor Cell Killing Assay

For OT-1 stimulation experiments, CD8+ OT-1 T-cells were isolated from the spleens of naive OT-1 mice using CD8+ MACS beads (Miltenyi) according to the manufacturer’s instructions. ImLECs were starved over night in Ham’s F12/DMEM + 1% FBS, pulsed with 1 ng/ml SIINFEKL peptide (AnaSpec) for 1 h, and washed three times with PBS. Subsequently, OT-1 and peptide-pulsed imLECs were cocultured for 24 h at a 10:1 ratio in the presence of 10 µg/ml PDL1-blocking antibody (clone 10F.9G2, Biolegend) or control rat IgG (Sigma). For analysis of T-cell activation, OT-1 cells were harvested and stained with rat anti-CD8-FITC (1:200, clone 53.6-7, BD), rat anti-PDL1-PE (1:200, clone MIH5, eBioscience), and rat anti-CD25-PerCP (1:200, clone PC61, Biolegend). Intracellular staining for IFN-g (1:200, clone XMG1.2 conjugated to APC, Biolegend) was done using the Cytofix/Cytoperm kit (BD) and fixable Zombi-NIR (BioLegend) was used for life/dead discrimination. For imLEC pre-blocking experiments, the PDL1-blocking antibody was incubated with the imLECs together with the SIINFEKL peptide pulse before washing and coculture with OT-1 cells. For tumor cell killing assays, OT-1 cells were stimulated with peptide-pulsed imLECs as described above and subsequently cocultured with B16F10 cells expressing ovalbumin at a 1:5 target:effector ratio for 8 h. Zombi-NIR was used to determine the ratio of dead and life tumor cells. All activation and killing assays were performed in triplicate. Data were acquired on a FACS CANTO and analyzed using FlowJo.

### Statistical Analyses

All statistical analyses were performed with GraphPad Prism 5 (GraphPad Software Inc.). All bars indicate mean + SD. Student’s *t*-test was used to compare two groups, one-way ANOVA with Tukey’s post-test was used to compare more than two groups, and two-way ANOVA with Bonferroni post-test was used to compare data grouped by two variables. A *p*-value <0.05 was considered statistically significant (indicated by asterisks).

## Author Contributions

LD designed and performed experiments, analyzed and interpreted the data, and wrote the manuscript. KI, TC, KK, and CH performed experiments, and analyzed and interpreted the data. MD designed experiments, interpreted data, and revised the manuscript.

## Conflict of Interest Statement

The authors declare that the research was conducted in the absence of any commercial or financial relationships that could be construed as a potential conflict of interest.
