# Supplementary material for: Tumor-Associated Lymphatic Vessels Upregulate PDL1 to Inhibit T-Cell Activation
Source: Front Immunol. 2017 Feb 3;8:66. doi: 10.3389/fimmu.2017.00066 (PMC5289955; doi:10.3389/fimmu.2017.00066)
Supplement: Supplementary file 1 [file Data_Sheet_1.PDF]

Supplementary Figure 1

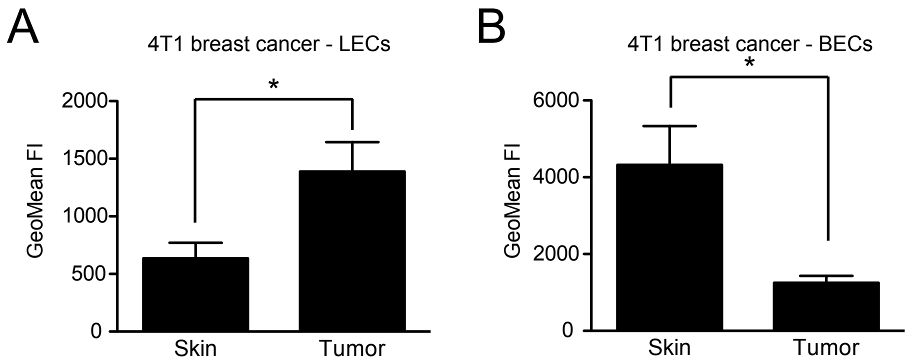

Supplementary Figure 1:
PDL1 expression in tumor-associated LECs and BECs is regulated early during tumor growth.
FACS analysis of tumor-associated LECs **(A)** and BECs **(B)** 8 days after 4T1 implantation. Similar to the 3 week timepoint, PDL1 expression is significantly induced in LECs, and significantly reduced in BECs (N=6 mice / group).
