# Supplementary material for: Tumor-Associated Lymphatic Vessels Upregulate PDL1 to Inhibit T-Cell Activation
Source: Front Immunol. 2017 Feb 3;8:66. doi: 10.3389/fimmu.2017.00066 (PMC5289955; doi:10.3389/fimmu.2017.00066)
Supplement: Supplementary file 2 [file Data_Sheet_2.PDF]

Supplementary Figure 2

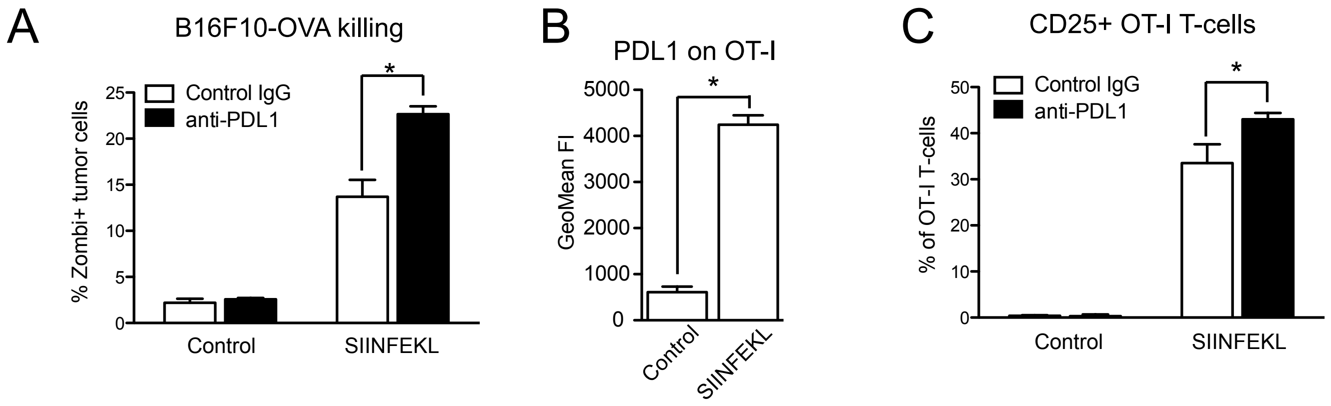

Supplementary Figure 2:
PDL1 inhibition on antigen-presenting imLEC increases OT-1 T-cell activation.
(A) Killing of B16F10-OVA cells in vitro by OT-1 cells pre-activated by SIINFEKL-presenting imLEC in the presence of PDL1 blocking antibodies or control IgG. **(B)** PD-L1 expression on OT-1 T-cells after co-culture with SIINFEKL-presenting imLEC. **(C)** Upregulation of CD25 on OT-1 T-cells after co-culture with imLEC presenting SIINFEKL peptide and pre-blocked with anti-PDL1.
